# Special Issue Physiological and Molecular Responses of Plants to Engineered Nanomaterials

**DOI:** 10.3390/nano14020151

**Published:** 2024-01-10

**Authors:** Marta Marmiroli, Elena Maestri

**Affiliations:** Department of Chemistry, Life Science and Environmental Sustainability, University of Parma, 43124 Parma, Italy; elena.maestri@unipr.it

## 1. Introduction

Engineered nanomaterials (ENMs), by definition materials with a size between 1 and 100 nm, are becoming an important part of the economy and thanks to their many fields of applications, from photovoltaic cells to fertilizers, are increasingly coming into contact with plants and with the environment in general. However, what the effects are of these novel materials, with particular characteristics due to, among other things, their size and reactivity, on plant physiology and molecular processes is still far from clear. In this issue, we aim to collect studies on this topic to elucidate the interconnection between ENMs and photosynthetic organisms.

## 2. Physiological and Molecular Responses of Plants to Engineered Nanomaterials

With the aim of collecting works on the topic of the physiological and molecular response of plants to ENMs, we collected 11 contributions, 7 of which are research articles and 4 are reviews.

The first contribution by Bruno Komazec and coworkers [1] regards the oxidative stress that the ENMs can cause, in this case, silver nanoparticles (AgNPs), widely used for their antimicrobial properties, on the microalgae *Chlorella vulgaris*. Algae were exposed to AgNPs stabilized with citrate and cetyltrimethylammonium bromide (CTAB) agents and to AgNO_3_. Algal response was investigated through the analysis of silver accumulation, ROS (reactive oxygen species) content, damage to biomolecules (lipids, proteins, and DNA), activity of antioxidant molecules, enzymatic and non-enzymatic, and changes in ultrastructure. The results showed that all treatments induced oxidative stress detrimental to algae. AgNP-CTAB showed the least toxic effect and caused the least oxidative damage. These results highlight the importance of surface-stabilizing agents in determining the phytotoxicity of AgNPs and the underlying mechanisms affecting aquatic organisms.

Another aquatic plant, a higher plant in this case, *Salvinia minima*, was studied in the interaction with coated and non-coated gold nanoparticles (Au NPs) by the group of Ndeke Mussee and coworkers [2]. No evidence of internalization was present; consequently, adsorption of Au NP accumulation by *S. minima* was thought to be the main mechanism of accumulation. Exposure of *S. minima* to Au NP in the long term did not inhibit plant biomass and growth rate, but the authors concluded that the formation of agglomerates on plant roots may block cell wall pores and alter the uptake of essential macronutrients affecting the overall ecological function.

The other five research papers are on the application of ENMs in agriculture, with two on wheat, two on tomato, and one on citrus.

In the first article by Ahmad Omar et al. [3], the positive effect of Se (SeNPs, 10 mg·L^−1^) nanoparticles was investigated against the damaging effects of drought and heat stresses on eight bread wheat (*Triticum aestivum*) genotypes. The foliar application of SeNPs improved plant growth, morpho-physiological and biochemical responses, the expression of stress-responsive genes in wheat seedlings, photosynthetic pigments, photosynthetic rate, gas exchange, and transpiration rate, and it enhanced drought and heat tolerance by increasing the activity of antioxidant enzymes and the expression level of stress-responsive genes *PIP1*, *LEA-1*, *HSP70*, and *HSP90*. It was concluded that several physio-biochemicals and gene expression attributes under drought and heat stress could be modulated in wheat genotypes by the foliar spray of SeNPs, potentially alleviating the adverse effects of drought and heat stresses.

The second article on the effect of nanomaterials on wheat is by Xiangning Huang and Arturo Keller [4], who employed metabolomics to understand the effects of Triton^TM^ X-100 surfactant (SA) and nanomaterials (NMs) on wheat (*Triticum aestivum*) at the molecular level. Leaves of wheat seedlings were foliar sprayed with different treatments: deionized water (DI), surfactant solution (SA), NM-surfactant suspensions (Cu(OH)_2_ NMs and MoO_3_ NMs), and ionic-surfactant solutions (Cu ions and Mo ions). The seedling leaves and roots were analysed via physiological parameters, nutrient distribution, and targeted metabolomics. Due to the low dissolution of Cu(OH)_2_ NMs in SA, minimal plant response was caused by this treatment, whilst given the high dissolution of MoO_3_ NMs, the corresponding high levels of Mo ions resulted in significant metabolite reprogramming (30+ metabolites dysregulated). Information gained from this study provides novel insights in relation to the use of surfactants to enhance the foliar application of nano agrochemicals.

The first paper on tomatoes, written by Baoshan Xing and coworkers [5], deals with the improvement of flavonoid content, which is very important from a nutraceutical point of view, in tomato fruit through the treatment with triiron tetrairon phosphate (Fe_7_(PO_4_)_6_) nanomaterials (NMs). They report that 50 mg kg^−1^ of Fe_7_(PO_4_)_6_ NMs, administered through the soil, was the optimal dose based on its outstanding performance on promoting tomato fruit flavonoid accumulation. Fe_7_(PO_4_)_6_ NMs entered the plant roots and promoted auxin (IAA) levels; they upregulated the expression of genes related to plasma membrane H^+^ ATPase, leading to root proton efflux and rhizosphere acidification, which consequently increased the concentrations of Mg, Fe, and Mn taken up by plants. Consequently, more photosynthate was produced and transported into fruits more rapidly, where it promoted flavonoid synthesis. Metabolomics and transcriptomics analyses of fruits revealed that Fe_7_(PO_4_)_6_ NMs regulated several important pathways and, finally, enhanced flavonoid biosynthesis. This study highlights the potential of NMs to improve fruit quality, in this case by enhancing flavonoids synthesis and accumulation.

The second paper on tomatoes, by Jason White, Jorge Gardea-Torresday and coworkers [6], studies Fe_3_O_4_, Fe_3_O_4_, MnFe_2_O_4_, ZnFe_2_O_4_, Zn_0.5_Mn_0.5_Fe_2_O_4_, Mn_3_O_4_, and ZnO nanomaterials (NMs), which were administered to tomato plants via foliar application to investigate their effects on the nutritional quality of the fruits. The plants grew for 135 days in a greenhouse, and the tomato fruits were harvested as they ripened. The lycopene and carotene content were reduced by almost all the treatments. However, the total phenolic compounds were increased by ZnFe_2_O_4_, Zn_0.5_Mn_0.5_Fe_2_O_4_, and ZnO, and the sugar content in the fruit was enhanced after exposure to Mn_3_O_4_ and ZnO. Beneficial and detrimental effects of various NMs on tomato fruit quality have been highlighted by this work and the need for caution in nanoscale applications during crop growth is advised.

Nanomaterials can also be useful against pests. This is the topic of the paper by Muhammad Ikram and coworkers [7], who studied the effect of selenium nanoparticles (SeNPs) to improve the health of Huanglongbing-infected ‘Kinnow’ mandarin plants. PCRs with specific primers were used to detect HLB disease in ‘Kinnow’ mandarin plants. The products were sequenced to identify *Candidatus* Liberibacter asiaticus (CLas). SeNPs were synthesized by using *Allium sativum* clove extract as a reducing, capping, and stabilizing agent and were characterized with various techniques. The results proved that 75 mg L^−1^ of SeNPs was most effective for improving chlorophyll, carotenoids, relative water content, membrane stability index, total soluble sugars, antioxidant enzymes, and other antioxidant molecules, and a general decrease in ROS was measured in HLB-infected ‘Kinnow’ mandarin plants as compared to untreated diseased plants. These results confirm that SeNP formulation could be a promising management strategy for treating HLB disease in citrus plants.

Three reviews deal on how nanomaterials can improve agricultural practice to face the future needs of this sector, while the last review reports on the negative environmental effects of Ag NPs, especially on aquatic systems and on algae.

The first review, written by Francisco Carmona and coworkers [8], deals with the possibilities offered by nanotechnology of designing and preparing novel alternative materials to conventional fertilizers, which are more readily absorbed by plant roots and therefore enhance nutrient use efficiency. In this context, great attention has been paid to calcium phosphate nanoparticles (CaP), particularly nanocrystalline apatite and amorphous calcium phosphate, as potential macronutrient nano-fertilizers with superior nutrient use efficiency to their conventional fertilizers. These nano-fertilizers have been functionalized with macronutrients like urea or nitrate to generate N-nano-fertilizers with more advantageous nitrogen-releasing profiles. The advances of this topic are reviewed and the challenges in the progress toward the real application of CaP as nano-fertilizers, involving several fields (i.e., agronomic or material science sectors), are highlighted and discussed.

The second review, written by Zishan Ahmad and coworkers [9], describes how nanoparticles and nanotechnology can play a significant role in plant growth, development, pathogen detection and crop protection, the delivery of genetic material, plant growth regulators and agrochemicals, and plant genetic engineering. They argue that agriculture has to face changes in climatic conditions, biotic and abiotic stresses that cause significant damages around the world, and the growing population demands. Nanotechnology can provide important tools to achieve a sustainable agricultural system. The aim of this review is to aid researchers to learn quickly how to use plant nanotechnology to improve agricultural production.

In the third review, written by Nosheen Akhtar and coworkers [10], it is discussed how the drastic changes in the climate and ecosystem, caused by natural or anthropogenic activities, have severely affected global crop production. They argue that this concern has raised the need to develop environmentally friendly and cost-effective strategies for keeping pace with the demands of the ever-growing population. Combining nanoparticles and biofertilizers produces nano biofertilizers which consist of biofertilizers encapsulated in nanoparticles. Biofertilizers consist of the preparation of plant-based carriers with beneficial microbial cells, while nanoparticles are microscopic particles that possess numerous advantages: silicon, zinc, copper, iron, and silver are the commonly used nanoparticles for the formulation of nano biofertilizer. There is only scant literature on the field application of biofertilizers, which is a strategy that if correctly managed can reduce the use of chemical fertilizer and make soil and crops healthy. The paper highlights the formulation and application of nano biofertilizers on various plant species and explains how nano biofertilizers can be of aid in improving the growth and development of plants. The review covers the role and status of nano biofertilizers in agriculture and the limitations of and future strategies for formulating effective nano biofertilizers.

The last review, by Renata Biba and coworkers [11], completes the cycle because it deals with the impact of ENMs on aquatic ecosystems. Silver nanoparticles (AgNPs) have been implemented in a wide range of commercial products, which are then released into aquatic and terrestrial systems without regulations. In this review, the authors attempt to provide an overview on how the use of different stabilizing coating agents can modulate AgNP-induced phytotoxicity related to growth, physiology, and gene and protein expression in terrestrial and aquatic plants and freshwater algae.

## 3. Future of the Understanding of the Physiological and Molecular Effects of Nanoparticles on Plants

Agriculture will surely be at the forefront of the studies that will aim at clarifying the effects of nanoparticles on plants. However, the study of their release in the terrestrial and aquatic environment will help to clarify their interaction, not only with higher plants, but also with marine photosynthetic organisms such as macro and microalgae.
